# The greater use of flavoured snus among ever-smokers versus never-smokers in Norway

**DOI:** 10.1186/s12954-020-00419-7

**Published:** 2020-10-16

**Authors:** Tord Finne Vedoy, Karl Erik Lund

**Affiliations:** Department Alcohol, Tobacco and Drugs, Norwegian Institute of Public Health, PO Box 222, 0213 Skøyen, Oslo, Norway

**Keywords:** Smokeless tobacco, Snus, Tobacco, Flavour, Regulation

## Abstract

**Background:**

Similar to the debate around e-cigarettes, an increase in snus use among Norwegian adolescents has prompted debate on whether flavour options in snus should be limited. To this end, we compared use of flavoured snus among snus users with different smoking status.

**Methods:**

Questions about flavoured snus use were included in an online omnibus study conducted from 2015 to 2019 (*N* = 65,445) that included 16,295 ever snus users (aged 15+). Current snus users (*N* = 9783) were asked “Do you usually use snus that has a flavouring (liquorice, mint, wintergreen, etc.)? Adjusted predicted probabilities and 95% confidence intervals (CI) were calculated from a logistic regression model.

**Results:**

Less than 25% of the snus users reported never having smoked. The overall probability of using flavoured snus was .45 (95% CI .44–.46), highest among daily (.51, 95% CI .47–.54) and former daily smokers (.50, 95% CI .48–.52), and lowest among never (.41, 95% CI .39–.43) and occasional smokers without any prior history of daily smoking (.41, 95% CI .38–.44). Use of flavoured products was higher among female snus users (*p* = .67, 95% CI .65–.69) compared to males (*p* = .35, 95% CI .34–.36), highest among the youngest age group, 15–24 years (*p* = .58, 95% CI .56–.60) and decreased with increasing age.

**Conclusion:**

Regulation that would ban or limit flavoured snus use may affect smokers—an at risk population—more than never smokers. The health authorities should be mindful of the real-world complexity governing potential harms and benefits of flavour restrictions on snus. A further assessment of flavour limitations should acknowledge that flavoured snus products also function as alternatives to cigarettes.

## Introduction

Cigarette smoking is a leading cause of mortality and causes an estimated 6.3 million deaths globally each year [1]. Use of snus—a low-nitrosamine Swedish type of oral tobacco—has no or very weak association with the diseases that combined cause the majority of smoking attributable mortality—lung cancer, respiratory diseases and cardiovascular diseases [2]. In 2012, the WHO Framework Convention on Tobacco Control (FCTC) advised Parties to regulate, by prohibiting or restricting, ingredients that may be used to increase tobacco product attractiveness [3]. Accordingly, the 2014 European Tobacco Product Directive (TPD) prohibits cigarettes and roll-your-own tobacco with a characterizing flavour other than tobacco [4]. In the USA, the Family Smoking Prevention and Control Act banned flavoured cigarettes (with the exemption of menthol) in 2009. Non-cigarette tobacco products, such as e-cigarettes and smokeless tobacco, have so far not been subject to equivalent regulation. However, California, Massachusetts, New Jersey, New York and Rhode Island, and dozens of US cities, have enacted policies ending the sale of flavoured e-cigarettes, and in Europe, the Dutch Government has recently proposed banning all non-tobacco flavours in e-cigarettes from 2021 [5, 6].

Snus has been banned in the EU since 1992. However, in Sweden (exempted from the ban), Norway (not an EU member) and the USA, multiple flavours of snus, including mint, whiskey, bergamot, liquorice and nuts, have been on the market for a long time [7]. In addition, a new type of portion-sized snus bags, branded as "white" or "all white", consisting of flavoured nicotine powder with no or very small amounts of tobacco, has recently emerged with fruit flavours like melon, blueberry, lime or apple. While the proportion of snus users that consume flavoured products is currently unknown in Norway and Sweden, the majority of snus users in the USA consume mentholated products [8].

In the case of Norway, the prevalence of daily snus use surpassed the prevalence of smoking around 2013 among adult men (16–74 years). Among young adults (16–24 years), this shift took place around 2006 among men and around 2011 among women [9]. The inverse correlation between the trends in smoking and snus use in the Norwegian population after 2000 has been described in several publications [10–12].

From 1999 to 2018, the market share of snus increased from 5 to 40% at the expense of cigarettes [13]. Among young adults (16–24 years old), the prevalence of daily snus use increased from 12 to 24% among men and from 1 to 16% among women in the period 2003 to 2017. In contrast, the prevalence of daily smoking among young adults decreased from 25 to 2% among women and from 26 to 5% among men in the same period [12].

The decrease in smoking prevalence is likely a result of persistent tobacco control efforts where Norway consistently ranks among the top five strictest countries in Europe [14]. The authorities' many measures to curb smoking may have incentivised smokers to look for alternative nicotine products. In fact, in Norway snus is by far the most used method in quit smoking attempts (after unassisted quitting) [15]. However, the rapid market shift may also partly be the result of industry driven product innovations. While cigarettes have remained mostly unchanged on the nicotine market, snus has been through several changes that have resulted in increased appeal—not only to smokers, but probably also to never-smokers [16]. Traditionally, snus was sold on the market as a standardized tobacco product in plain cardboard boxes, without any flavour descriptor and typically containing 50 g of loose snus. Over time, many new snus brands emerged on the marketplace, often with a variety of sub-brands with different nicotine concentrations. Moreover, the snus package became more colourful and cans became increasingly varied with regards to shape, size and weight, most often produced in plastic or tin and typically containing snus in a variety of user-friendly sachets (large, slim, super-slim, mini) made of cellulose. This ended in July 2017 when plain packaging of cigarettes and snus was introduced. Furthermore, innovations in the manufacturing process has over time reduced the formation of carcinogenic tobacco-specific nitrosamines (TSNAs) and polycyclic hydrocarbons (PAHs) in snus [15].

### Flavour limitations

Similar to the debate around e-cigarettes in the USA, the increase in snus use among Norwegian adolescents has prompted a debate about whether limiting flavour options in snus should be a part of a future strategy to reduce tobacco related harm. Proponents claim that flavoured products mask unpleasant taste and make products more palatable. They refer to literature suggesting that flavoured smokeless tobacco (ST) products appeal to youth [16–22] and may increase curiosity and willingness to try snus [23].

In historic tobacco industry documents, flavoured ST were referred to as “starter products”, a strategy to target inexperienced users [24, 25]. Proponents of flavour restrictions also cite recent US studies [18, 19] showing that most adolescent ST users use flavoured products. In one US study of young adult ST users, where 80% reported use of flavoured products, 60% predicted they would discontinue their use of smokeless tobacco if products were not flavoured [26]. According to the proponents, restricting flavours in ST products might therefore have the potential to reduce the prevalence of young people’s tobacco use.

On the other hand, opponents of limiting flavoured products argue that flavoured non-combustible products, such as snus, offer a harm reduction pathway to smokers (or users who would otherwise smoke), which produces an overall net benefit to public health. No such benefit applies in the case of combustible cigarettes. A different approach should therefore be used to analyse the public health impacts of non-combustible products and to define appropriate policy regarding flavours. According to the opponents, combustible and non-combustible flavoured products should not be lumped together in policy considerations, given the pronounced differences in risk and the opportunity to reduce health risks among people who would otherwise smoke. Opponents typically refer to studies showing that smokers also have strong preferences for flavoured smokeless tobacco products over tobacco-dominant flavours [27–29], and that these products are not associated with greater dependence or increased exposure to nicotine or carcinogens [28]. According to the opponents, restricting flavours in non-combustible products might lower the intentions to replace smoking with a harm reducing uptake of nicotine, as suggested in several studies [30–34].

Given that flavours may affect the potential of snus to be both a substitute and a complement to cigarette smoking, designing a health optimal flavour regulation for snus is not straightforward. Ideally, a justification for an intervention on snus flavours should demonstrate that this would in fact be appropriate for the protection of public health, and that it is reasonable to expect that the benefits will outweigh the harms.

### Aims

To date, there has been limited research reporting on the use of flavoured snus in Norway. A valuable input to lawmakers and regulators would be to identify the extent to which flavoured snus is used, and whether use of flavoured products differ among snus users with different experiences of cigarette smoking. Therefore, the aims of this study were to (1) decompose snus use according to smoking status in order to (2) compare the probability of using flavoured snus among snus users with different smoking patterns. The results will be discussed within a public health framework to consider potential costs and benefits from flavour restrictions on snus.

## Methods

### Material

Questions about cigarette and snus use were included in a weekly online omnibus survey in the period February 2015–December 2019. The online survey was administrated by the international research agency Ipsos on behalf of the Norwegian Institute of Public Health, and included a total of 65,445 persons (aged 15+) in this period. For 20% of the sample, primarily those above the age of 60, an invitation to the survey was made via e-mail to members of a large web-panel. People were recruited to this web panel after having participated in previous nationally representative population surveys, carried out by telephone, post, or personal interviews, and had agreed to receive future invitations to participate in surveys by e-mail. Self-recruitment to the panel was not possible and none of the panellists were paid for their participation. The remaining 80% were contacted via text messages on telephone numbers drawn from a copy of the national population register, provided by Bisnode. The study did not contain personally identifiable data and in accordance with the Norwegian Health Research Act, the project did not need approval from Regional Committees for Medical and Health Research Ethics.

The sample was randomly drawn from Bisnodes population register, but monitored to match national representativeness on demographic variables of sex, region, urbanity and age. If an insufficient number of persons responded in some population segments, additional e-mails were sent to new respondents in that group until a sample reflective of the study population was achieved (quota sampling). To avoid double responses, respondents were removed from the list of potential panellists for future studies. The questionnaire was adapted to fit computer-, smartphone- and tablet formats.

### Measures

Tobacco product use was assessed by applying identical questions and response categories for smoking and snus use. The wording of the question was: “Which category best describes your current (smoking/snus use) status?” The six mutually exclusive response categories were: (1) *current daily user*, (2) *current occasional user, former daily user*, (3) *current occasional user, never daily user*, (4) *former daily user*, (5) *former occasional user*, (6) *never user* (Table 1).

**Table 1:** Descriptive statistics for current, former and never snus users

	Current snus users (*N* = 9515)	Former snus users (6482)	Never snus users (48,699)
	% (95% CI)	% (95% CI)	% (95% CI)
*Snus use status*			
Daily snus user	70.6 (69.6–71.5)		
Occasional snus user, former daily user	12.2 (11.6–12.9)		
Occasional snus user, never daily user	17.2 (16.5–18.0)		
Former daily snus user		46.6 (45.4–47.9)	
Former occasional snus user		53.4 (52.1–54.6)	
Never used snus			
*Smoking status*			
Daily smoker	9.8 (9.2–10.3)	9.2 (8.5–9.9)	7.9 (7.7–8.1)
Occasional smoker, former daily smoker	12.0 (11.3–12.6)	4.6 (4.1–5.2)	3.0 (2.8–3.1)
Occasional smoker, never daily smoker	9.6 (9.0–10.2)	3.9 (3.4–4.4)	2.1 (1.9–2.2)
Former daily smoker	27.2 (26.3–28.1)	33.5 (32.3–34.6)	21.3 (21.0–21.7)
Former occasional smoker	19.0 (18.2–19.7)	25.3 (24.2–26.3)	13.6 (13.3–13.9)
Never smoked	22.5 (21.7–23.4)	23.5 (22.5–24.5)	52.1 (51.7–52.5)
*Sex*			
Men	68.0 (67.1–68.9)	65.3 (64.2–66.5)	43.8 (43.4–44.2)
Women	32.0 (31.1–32.9)	34.7 (33.5–35.8)	56.2 (55.8–56.6)
*Age*			
15–24*	22.5 (21.6–23.3)	13.9 (13.0–14.7)	9.1 (8.8–9.3)
25–34	29.7 (28.7–30.6)	30.1 (29.0–31.2)	12.7 (12.4–13.0)
35–44	26.8 (25.9–27.7)	28.8 (27.7–29.9)	20.5 (20.1–20.9)
45–54	14.2 (13.5–14.9)	16.2 (15.3–17.1)	19.8 (19.5–20.2)
55–64	5.0 (4.6–5.4)	7.2 (6.6–7.9)	17.0 (16.7–17.3)
65–74	1.8 (1.5–2.1)	3.2 (2.8–3.6)	16.6 (16.3–17.0)
75+	0.1 (0.0–0.2)	0.6 (0.4–0.8)	4.3 (4.2–4.5)
Mean age	35 (34.7–35.2)	38.0 (37.7–38.3)	48.5 (48.3–48.6)
*Education*			
Primary	5.6 (5.2–6.1)	4.7 (4.2–5.3)	7.3 (7.0–7.5)
Secondary	38.6 (37.6–39.6)	32.8 (31.7–34.0)	30.9 (30.5–31.3)
Tertiary (≤ 3 years)	39.1 (38.1–40.1)	40.9 (39.7–42.1)	40.2 (39.7–40.6)
Tertiary (≥ 4 years)	16.7 (15.9–17.4)	21.5 (20.5–22.5)	21.7 (21.3–22.0)

Percentages (%) and 95% confidence intervals (95% CI)

^a^Minimum age for buying tobacco in Norway is 18 years. Respondents below the age of 18 made up 21% of the 15–24 year age group. There was no abrupt change in snus use or smoking between respondents below and above 18-years

Among a total of 16,295 ever snus users (categories 1–5), current snus users (categories 1–3, *N* = 9783) were asked *Do you usually use snus that has a flavouring* (e.g. *liquorice, mint, wintergreen *etc.)? Response options were *yes*, *no* and *do not know*. Respondents answering *do not know* (*n* = 229) or who did not answer the question (*n* = 1), in total 230 (2.3%), were excluded from the analyses.

Crosstab analyses stratified snus use and cigarette smoking status into 36 categories of tobacco product use, including 18 categories of current snus users of which nine categories were dual users of snus and cigarettes, six categories were former smokers and three categories were never smokers (Table 2).

**Table 2:**
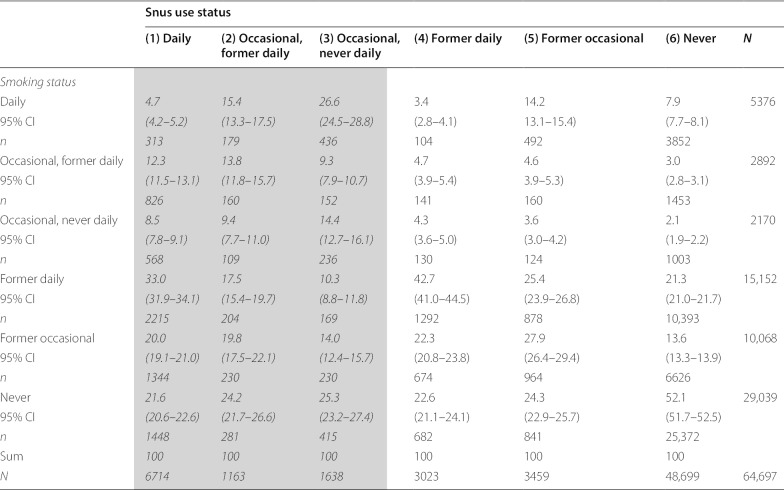
Distribution of smoking status (in percent) among current snus users (in gray), former snus users and never users of snus

Respondents’ socio-economic position was measured using four categories of education: completion of 9 years of compulsory education (primary), at least 3 years of high-school education (secondary), a Bachelor's degree or a Master’s degree. Age was recoded into 10-year age groups (15–24, 25–34, …, 65–74, 75+).

### Analysis

To examine the use of flavoured snus among current snus users with various smoking patterns, we constructed two nested logistic regression models in Stata 15 [35] with a dichotomous measure of flavoured snus use as the dependent variable and snus use status, smoking status, education, gender and age as independent variables. Model 1 included all independent variables, while Model 2 included all variables and an interaction between snus use status and smoking status. Model sample size, coefficients (logits) and Akaike information criterion (AIC)/Bayesian information criterion (BIC) are shown in Additional file 1: Appendix 1. Among all current snus users (*N* = 9554), 9515 (99.6%) had information on all variables.

The significance of the interactions between smoking and snus use was tested with a Wald test (testparm command) in Stata 15. The test rejected the null, i.e. that all pairwise combinations of snus use and smoking were equal to 0 (Chi^2^ = 31.2, *p* < 0.001). AIC and BIC did not provide support for the same model. Consequently, we chose Model 2 for the subsequent analyses, because of the statistically significant interaction.

From Model 2 we calculated average adjusted probabilities [hereafter called probabilities (*p*)] and 95% confidence intervals (CI) of using snus with flavour for all values of the independent variables, using the margins command. We employed Bonferroni correction when comparing average adjusted probabilities.

## Results

Distribution of sex, education, snus use, smoking characteristics and age for current, former and never snus users are described in Table 1. In general, current and former snus users were most often young adult men with secondary or lower tertiary education who were either former daily or never smokers.

### Smoking among snus users

The prevalence of daily, occasional, former and never smokers among all subgroups of snus users are shown in Table 2. Results for the three categories of current snus users (1–3) that answered the question about flavoured snus are marked in grey.

In all five categories of ever snus use, never smokers comprised 25.3% or less. 45.3% of daily snus users reported to be former daily smokers who had either quit all smoking (33.0%) or who still smoked occasionally (12.3%). 20.0% of daily snus users reported to be former occasional smokers and 8.5% reported occasional smokers who had never smoked daily. Only 4.7% of daily snus users were also daily smokers, significantly lower than the proportion of daily smokers among non-users of snus (7.9%).

There was substantial variation in smoking between sub-groups of snus users. For example, among respondents who used snus occasionally, but never daily, 26.6% smoked daily. Among respondents who used snus occasionally, but had used snus on a daily basis previously, the corresponding figure was 15.4%. Likewise, the percentage of former daily smokers among daily snus users was 33.0% compared to 10.3% among occasional snus users that had never used snus daily.

### Use of flavoured snus

Adjusted predicted probabilities from the multivariate logistic regression model (Model 2) showed that the overall probability (*p*) of using snus with flavour among all current snus users was 0.45 (95% CI 0.44–0.46). The probability was higher among women (*p* = 0.67, 95% CI 0.65–0.69) compared to men (*p* = 0.35, 95% CI 0.34–0.36). With regard to age, popularity of flavours was highest among the youngest age group, 15–24 years (*p* = 0.58, 95% CI 0.56–0.60) and decreased with increasing age. Snus users that had completed 9 years of compulsory education had a higher probability of using snus with flavour (*p* = 0.49, 95% CI 0.45–0.53) compared to respondents with a Master’s degree or above (*p* = 0.41, 95% CI 0.39–0.44) (Additional file 1: Appendix 2).

**Fig. 1:**
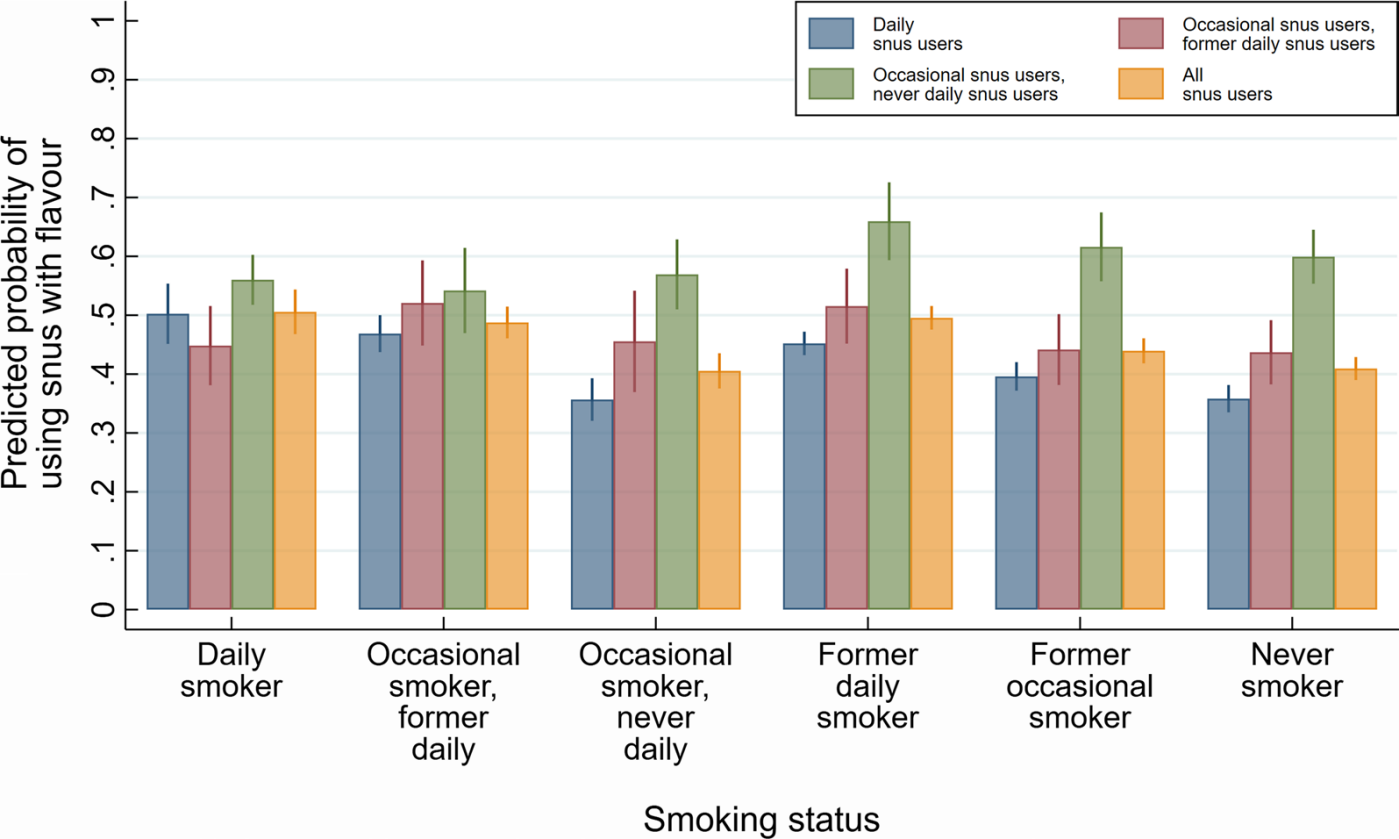
Adjusted predicted probabilities and 95% confidence intervals of using snus with flavour among six groups of current snus users defined by their smoking status

Figure 1 shows the probabilities of using flavoured snus among current snus users with different smoking patterns, controlling for sex, age, and education. The complete table of probabilities with Bonferroni corrected tests of differences are shown in Additional file 1: Appendix 2.

If we look at current snus users combined (yellow bars), results show that use of flavoured snus was highest among daily smokers (0.51, 95% CI 0.47–0.54) and former daily smokers (0.50, 95% CI 0.48–0.52) and lowest among never smokers (0.41, 95% CI 0.39–0.43) and occasional smokers without any prior history of daily smoking (0.41, 95% CI 0.38–0.44).

Within each of the six categories of smoking status in Fig. 1, we observe some variations in use of flavoured snus across snus use status. For example, in both groups of former smokers, occasional (but never daily) snus use was associated with significantly higher use of flavoured products. Also among never smokers, occasional (but never daily) snus use was associated with higher use of snus with flavourings.

## Discussion

Our study has shown that the majority of snus users (at least 75%)—whether current or former – were current or former smokers (ever smokers). This finding is consistent with Norwegian studies based on other data sets [36–38]. However, the novel finding from our study is that daily smokers and former daily smokers were more likely to use flavoured snus products compared to snus users without any prior history of smoking. A hypothetical ban or limitation of flavoured snus might then come to affect smokers more than never smokers. Thus, the belief that the primary purpose and consequence of restricting flavours in snus is to demotivate current non-users from future nicotine use might be simplistic. Regulation that would reduce the diversity and subsequent appeal of snus may lead to the unintended consequence of deterring smokers—an at-risk population—from a beneficial product switch.

On the other hand, we also observed that among subgroups of former smokers and never smokers, non-daily use of snus was associated with higher use of flavoured products. This might mean that snus users with an episodic association with the snus market have a preference for using products with non-tobacco flavourings.

### Relative risk between smoking and snus use

Swedish snus—while not being risk-free—has been acknowledged by the scientific community to be at the lower end of the risk scale of smokeless tobacco products [39–41]. A strong epidemiological evidence base shows no associations with lung cancer or respiratory diseases, and very weak, if any, association with cardiovascular diseases [2, 41], diseases that combined cause 2/3 of the smoking attributable mortality [42]. Moreover, there is no evidence that use of snus is associated with any major health hazard that does not also arise from tobacco smoking [2]. Medical expert committees assess the overall health risk from use of Swedish snus to be minor (below 10%) when compared to the risk from smoking [43–46].

In October 2019, the US Food and Drug Administration (FDA) authorized one manufacturer of Swedish snus to market products with the claim “Using General Snus instead of cigarettes puts you at a lower risk of mouth cancer, heart disease, lung cancer, stroke, emphysema, and chronic bronchitis” [47]. The authorization was based on a review of scientific evidence demonstrating that snus—as actually used by consumers—would significantly reduce harm and the risk of tobacco-related disease to individual tobacco users and benefit the health of the population as a whole.

Still, some commentators are concerned that flavours may encourage snus use among never-smokers to such a degree that the combined negative health effect on the population increases, even when taking into account the reduced risk for smokers who swap cigarettes for flavoured snus.

### Risk-use equilibrium

To evaluate the possible problems caused by flavours in snus, it is helpful to consider what might be called the risk/use equilibrium [48]. Given the relatively low excess risk for never smokers who take up snus, and given the epidemiological verified large risk reduction for smokers who switch to snus, the number of never-smokers taking up snus prompted by flavours must be implausibly large to balance out the health gain from the smokers who make a flavour-driven product switch to snus. For net harm to occur, an epidemiological modelling study estimated that 14–25 ex-smokers would have to start using snus to offset the health gain from every smoker who switched to snus rather than continuing to smoke. Likewise, 14–25 people who have never smoked would need to start using snus to offset the health gain from every new tobacco user who used snus rather than smoking [49].

In the present study, the absolute number of daily smokers was 5376, or 8.3% of the total sample (95% CI 8.1–8.5) (Table 2). The absolute number of daily smokers who had never used snus was 3852, or 6.0% of the total sample (95% CI 5.8–6.1), almost three times higher that the number of daily snus users who had never smoked (*n* = 1448). This proportion would be similar even if we included occasional users of the two products. At the same time, respondents who had never used snus or smoked made up the largest group (*n* = 25,372 or 39.2% of the total sample, 95% CI 38.8–39.6). In this segment of potential snus users, susceptibility to flavoured products would contribute negatively to public health. However, according to our study—and several others—the largest reservoir of potential snus users have until now been smokers—not never smokers [36–38].

Given that flavoured snus use was observed to be most prevalent in the youngest age group, one objection could be that flavours increase snus use among young adults. If that were to occur, the net public health gains from adult smokers switching from cigarettes to flavoured snus could in part be undermined. It has been claimed that prior use of non-combustible tobacco products increases the risk of subsequent uptake of cigarette smoking [50]. Whether this association is a result of a causal mechanism, of common liabilities or unaddressed residual confounding has been a matter of dispute [51–54]. Anyway, if a causal association between snus use and subsequent smoking really exists, ecological trend data indicates that this association must have been dwarfed by other factors protecting against smoking uptake. Because at the population level, an inverse correlation between snus use and smoking has been observed among young adults in Norway [10], and recently also between vaping and smoking in the USA [55] and in New Zealand [56]. Thus, lending support to the "diversion theory" hypothesizing that snus/e-cigarettes might deter cigarette smoking by diverting "high-risk" individuals to snus/e-cigarettes from combustible cigarettes.

In our survey, the percentage of young adult daily smokers (aged 15–24 years) who had initiated their tobacco product use with snus was 20.8% (95% CI 16.8–25.4). Thus, a potential “gateway” mechanism from snus use to subsequent smoking could exist within a relatively small segment. But, snus users and smokers might have common liabilities, and separating a causal effect from residual confounding by common liabilities is methodologically challenging. Some of those who transit from snus to cigarettes would perhaps have started to smoke anyway—without any prior use of snus.

Thus, based on current knowledge of (a) the moderate risk of snus use relative to non-use, (b) the huge risk difference between snus use and smoking, (c) the overrepresentation of ever smokers relative to never smokers in the snus user population and (d) the relatively higher prevalence in the use of flavoured snus among ever daily smokers as compared to never smokers, there might be a danger that restrictions on flavoured snus—depending on the scope and type—can result in a net loss to public health. Health authorities should at least take this possible outcome into consideration.

### What should justify flavour restrictions?

Two justifications for flavour restrictions of snus would be to (1) eliminate properties of the flavour chemical that are harmful to health, or (2) eliminate any characterizing flavour that would initiate snus use among adolescents who in the absence of theses flavours would have remained tobacco-free (while taking into account that these flavours also may prompt smokers switch to snus). While the first should be a straightforward matter of toxicological testing, the second raises the question of how to identify a subset of flavours that could be defined as especially appealing to adolescents.

One option would be to focus on those brands that have the greatest proportion of sales to younger people. However, unless preferences are uniform across all age-bands, there will always be a category that has a higher youth uptake. The question is how pronounced should the bias towards youth sales be before the flavour becomes a concern for regulation.

Prior to an implementation of flavour limitations, policy makers should try to prove that the presence of certain flavours exert such a powerful attraction that they in fact create a change in behaviour. A recent synthesis identified important gaps in the literature as to how flavouring might influence patterns of tobacco use [20]. If flavours have the ability to change behaviour, policy makers should apply the risk-use equilibrium approach to balance the anticipated health gain in the segment of never smokers who will refrain from snus use due to limitation of flavours, against the anticipated health loss in the segment of smokers who will refrain from swapping cigarettes for snus due to flavour limitations.

### Strenghts and limitations

The findings in this study are subject to some limitations. Firstly, the self-reported nature of the survey could lead to misclassification of flavoured product use status. Our measure provided the respondents with examples of flavoured snus products (*liquorice, mint, wintergreen *etc*.*). However, far from all flavoured products come with descriptors informing the consumer of its flavour, and given that flavour perception is subjective, the accuracy of our measure can be questioned. Furthermore, all snus products, including those with taste of tobacco, have in some way or another been flavoured during manufacturing (e.g. with sugar). A more detailed description of the preferred flavours would have increased the validity of our results, and illustrate the need of ways of representing information that improve research on flavoured ST. However, we have no reason to believe there should be a difference in reporting use of flavoured snus between smokers and never-smokers.

Secondly, self-reported data on a socially deviant behaviour like smoking (and to a lesser degree snus use) might lead to misclassification due to social desirability bias. Thirdly, underrepresentation of respondents with primary education reduce the representativeness of the results. Lastly, being cross-sectional, the data do not enable us to determine the causal effects of smoking status on the use of flavoured snus. Further studies should examine the effect of flavoured snus use on both smoking cessation and snus initiation in different subgroups of smokers and snus users.

The strength of the study is the large sample of snus users, the consistent wording of the questions measuring smoking and snus use, and the very detailed decomposition of tobacco user status into 36 categories. With regards to the representativeness of smoking and snus use, we compared figures from the present dataset with a comparable population (16–79 year olds in the years 2015–2019) from a nationally representative survey conducted annually by Statistics Norway—a governmental body responsible for official statistics. The prevalence of current snus use was 15% in both surveys. The corresponding figures for current smoking was 16% in the current survey and 19% in the nationally representative survey.

To conclude, our study has shown that in Norway, use of flavoured snus is more prevalent among current and former daily smokers compared to never-smokers. In this context, the health authorities should be mindful of the real-world complexity governing potential harms and benefits of flavour restrictions on snus. A further assessment of flavour limitations should consider the possibility that flavoured snus products also function as alternatives to cigarettes (the main contributor to tobacco-related harm) and that the majority of current snus users are ever-smokers.

## Supplementary information

**Additional file 1**. Beta coefficients and model fit for Model 1 and Model 2. Adjusted probabilities of using snus with flavour and 95% confidence intervals (CI) in all groups from Model 2. Bonferroni corrected tests of differences.

## Data Availability

The datasets used during the current study are available from the corresponding author on reasonable request.

## Abbreviations

AIC: Akaike information criterion
BIC: Bayesian information criterion
CI: Confidence interval
EU: European Union
FCTC: Framework Convention on Tobacco Control
FDA: US Food and Drug Administration
PAH: Polycyclic aromatic hydrocarbons
ST: Smokeless tobacco
TPD: Tobacco product directive
TSNA: Tobacco specific nitrosamines
US: United States
WHO: World Health Organization
