# Supplementary material for: The greater use of flavoured snus among ever-smokers versus never-smokers in Norway
Source: Harm Reduct J. 2020 Oct 16;17:76. doi: 10.1186/s12954-020-00419-7 (PMC7568365; doi:10.1186/s12954-020-00419-7)
Supplement: Supplementary file 1 — Additional file 1. Beta coefficients and model fit for Model 1 and Model 2. Adjusted probabilities of using snus with flavour and 95% confidence intervals (CI) in all groups from Model 2. Bonferroni corrected tests of differences. [file 12954_2020_419_MOESM1_ESM.docx]

Appendix 1: Beta coefficients and model fit for Model 1 and Model 2

|  | Model 1* | | | Model 2** | | |
| --- | --- | --- | --- | --- | --- | --- |
| **Dependent variable: Using Snus with flavour** | b | se | p | b | se | p |
| Daily smoker (reference category (ref.)) | 0.00 | . | . | 0.00 | . | . |
| Occasional smoker, former daily smoker | 0.15 | 0.10 | 0.13 | -0.16 | 0.14 | 0.27 |
| Occasional smoker, never daily smoker | -0.26 | 0.11 | 0.01 | -0.70 | 0.15 | 0.00 |
| Former daily smoker | 0.15 | 0.09 | 0.08 | -0.23 | 0.13 | 0.07 |
| Former occasional smoker | -0.11 | 0.09 | 0.23 | -0.50 | 0.14 | 0.00 |
| Never smoker | -0.25 | 0.09 | 0.01 | -0.69 | 0.14 | 0.00 |
| Daily snus user (reference category (ref.)) | 0.00 | . | . | 0.00 | . | . |
| Occasional snus user, former daily snus user | 0.25 | 0.07 | 0.00 | -0.26 | 0.20 | 0.19 |
| Occasional snus user, never daily snus user | 0.67 | 0.07 | 0.00 | 0.08 | 0.16 | 0.60 |
| Men (ref.) | 0.00 | . | . | 0.00 | . | . |
| Women | 1.39 | 0.05 | 0.00 | 1.39 | 0.05 | 0.00 |
| 15-24 (ref.) | 0.00 | . | . | 0.00 | . | . |
| 25-34 | -0.50 | 0.07 | 0.00 | -0.49 | 0.07 | 0.00 |
| 35-44 | -0.79 | 0.07 | 0.00 | -0.78 | 0.07 | 0.00 |
| 45-54 | -0.92 | 0.08 | 0.00 | -0.91 | 0.08 | 0.00 |
| 55-64 | -1.29 | 0.12 | 0.00 | -1.27 | 0.12 | 0.00 |
| 65-74 | -1.48 | 0.20 | 0.00 | -1.46 | 0.20 | 0.00 |
| 75+ | -0.87 | 0.70 | 0.22 | -0.82 | 0.70 | 0.24 |
| Primary (ref.) | 0.00 | . | . | 0.00 | . | . |
| Secondary | -0.06 | 0.10 | 0.54 | -0.06 | 0.10 | 0.55 |
| Bachelor’s degree | -0.21 | 0.10 | 0.05 | -0.21 | 0.10 | 0.04 |
| Master’s degree or above | -0.34 | 0.11 | 0.00 | -0.35 | 0.11 | 0.00 |
| Daily smoker & Daily snus user (ref.) |  |  |  | 0.00 | . | . |
| Daily smoker & Occasional snus user, former daily snus user (ref.) |  |  |  | 0.00 | . | . |
| Daily smoker & Occasional snus user, never daily snus user (ref.) |  |  |  | 0.00 | . | . |
| Occasional smoker, former daily smoker & Daily snus user (ref.) |  |  |  | 0.00 | . | . |
| Occasional smoker, former daily smoker & Occasional snus user, former daily snus user |  |  |  | 0.49 | 0.27 | 0.07 |
| Occasional smoker, former daily smoker & Occasional snus user, never daily snus user |  |  |  | 0.07 | 0.25 | 0.78 |
| Occasional smoker, never daily smoker & Daily snus user (ref.) |  |  |  | 0.00 | . | . |
| Occasional smoker, never daily smoker & Occasional snus user, former daily snus user |  |  |  | 0.73 | 0.30 | 0.02 |
| Occasional smoker, never daily smoker & Occasional snus user, never daily snus user |  |  |  | 0.74 | 0.23 | 0.00 |
| Former daily smoker & Daily snus user (ref.) |  |  |  | 0.00 | . | . |
| Former daily smoker & Occasional snus user, former daily snus user |  |  |  | 0.55 | 0.25 | 0.03 |
| Former daily smoker & Occasional snus user, never daily snus user |  |  |  | 0.72 | 0.24 | 0.00 |
| Former occasional smoker & Daily snus user (ref.) |  |  |  | 0.00 | . | . |
| Former occasional smoker & Occasional snus user, former daily snus user |  |  |  | 0.47 | 0.25 | 0.06 |
| Former occasional smoker & Occasional snus user, never daily snus user |  |  |  | 0.77 | 0.22 | 0.00 |
| Never smoker & Daily snus user (ref.) |  |  |  | 0.00 | . | . |
| Never smoker & Occasional snus user, former daily snus user |  |  |  | 0.64 | 0.25 | 0.01 |
| Never smoker & Occasional snus user, never daily snus user |  |  |  | 0.88 | 0.20 | 0.00 |
| Constant | -0.01 | 0.13 | 0.95 | 0.36 | 0.16 | 0.02 |
| aic | 11612 | | | 11601 | | |
| bic | 11741 | | | 11801 | | |
| N | 9515 | | | 9515 | | |

* Stata code: logit flavour i.smoking i.snus i.sex i.age_10 i.education_4

** Stata code: logit flavour i.smoking##i.snus i.sex i.age_10 i.education_4

Appendix 2: Adjusted predicted probabilities of using snus with flavour and 95% confidence intervals (CI) in all groups from Model 2. Bonferroni corrected tests of differences.

|  | Margin | Bonferroni  95% CI | | Bonferroni Groups* |
| --- | --- | --- | --- | --- |
| **Smoking status** |  |  |  |  |
| Daily smoker | 0.506 | 0.468 | 0.544 | C |
| Occasional smoker, former daily smoker | 0.488 | 0.461 | 0.515 | BC |
| Occasional smoker, never daily smoker | 0.405 | 0.375 | 0.435 | A |
| Former daily smoker | 0.496 | 0.476 | 0.516 | C |
| Former occasional smoker | 0.439 | 0.418 | 0.461 | AB |
| Never smoker | 0.409 | 0.390 | 0.429 | A |
| **Snus use status** |  |  |  |  |
| Daily snus user | 0.425 | 0.414 | 0.437 |  |
| Occasional snus user, former daily snus user | 0.477 | 0.449 | 0.505 |  |
| Occasional snus user, never daily snus user | 0.575 | 0.547 | 0.602 |  |
| **Sex** |  |  |  |  |
| Men | 0.351 | 0.339 | 0.362 |  |
| Women | 0.669 | 0.652 | 0.686 |  |
| **Age** |  |  |  |  |
| 15-24 | 0.577 | 0.555 | 0.600 | D |
| 25-34 | 0.469 | 0.452 | 0.487 | C |
| 35-44 | 0.407 | 0.389 | 0.426 | B |
| 45-54 | 0.380 | 0.355 | 0.405 | AB |
| 55-64 | 0.308 | 0.268 | 0.348 | A |
| 65-74 | 0.274 | 0.207 | 0.341 | A |
| 75+ | 0.398 | 0.111 | 0.685 | ABCD |
| **Education** |  |  |  |  |
| Primary | 0.487 | 0.447 | 0.526 | BC |
| Secondary | 0.474 | 0.458 | 0.489 | C |
| Bachelor’s degree | 0.443 | 0.428 | 0.458 | AB |
| Master’s degree or above | 0.412 | 0.389 | 0.436 | A |
| **Smoking status X snus use status** |  |  |  |  |
| Daily smoker & Daily snus user | 0.510 | 0.459 | 0.561 | DE |
| Daily smoker & Occasional snus user, former daily snus user | 0.453 | 0.386 | 0.520 | ABCD |
| Daily smoker & Occasional snus user, never daily snus user | 0.528 | 0.484 | 0.571 | DE |
| Occasional smoker, former daily smoker & Daily snus user | 0.476 | 0.445 | 0.507 | CD |
| Occasional smoker, former daily smoker & Occasional snus user, former daily snus user | 0.525 | 0.453 | 0.597 | CDE |
| Occasional smoker, former daily smoker & Occasional snus user, never daily snus user | 0.509 | 0.435 | 0.583 | BCDE |
| Occasional smoker, never daily smoker & Daily snus user | 0.364 | 0.327 | 0.400 | AB |
| Occasional smoker, never daily smoker & Occasional snus user, former daily snus user | 0.460 | 0.375 | 0.546 | ABCDE |
| Occasional smoker, never daily smoker & Occasional snus user, never daily snus user | 0.537 | 0.476 | 0.598 | DE |
| Former daily smoker & Daily snus user | 0.459 | 0.439 | 0.479 | CD |
| Former daily smoker & Occasional snus user, former daily snus user | 0.520 | 0.457 | 0.583 | CDE |
| Former daily smoker & Occasional snus user, never daily snus user | 0.631 | 0.561 | 0.700 | E |
| Former occasional smoker & Daily snus user | 0.403 | 0.379 | 0.428 | ABC |
| Former occasional smoker & Occasional snus user, former daily snus user | 0.447 | 0.387 | 0.506 | ABCD |
| Former occasional smoker & Occasional snus user, never daily snus user | 0.585 | 0.525 | 0.646 | DE |
| Never smoker & Daily snus user | 0.365 | 0.342 | 0.388 | A |
| Never smoker & Occasional snus user, former daily snus user | 0.442 | 0.388 | 0.496 | ABCD |
| Never smoker & Occasional snus user, never daily snus user | 0.568 | 0.521 | 0.616 | DE |
| **Over all margin (constant)** | **0.452** | **0.443** | **0.461** |  |

* Between snus users within each variable or interaction between variables. Margins sharing a letter in the group label are not significantly different at the 5% level.
